# Estimation of Premature Deaths From Lack of Access to Anti-HER2 Therapy for Advanced Breast Cancer in the Brazilian Public Health System

**DOI:** 10.1200/JGO.2016.005678

**Published:** 2016-07-20

**Authors:** Márcio Debiasi, Tomás Reinert, Rafael Kaliks, Gilberto Amorim, Maira Caleffi, Carlos Sampaio, Gustavo dos Santos Fernandes, Carlos H. Barrios

**Affiliations:** **Márcio Debiasi** and **Carlos H. Barrios**, Latin American Cooperative Oncology Group; Márcio Debiasi, Hospital São Lucas da Pontificia Universidade Católica do Rio Grande do Sul; **Márcio Debiasi** and **Tomás Reinert**, Hospital do Câncer Mãe de Deus; Maira Caleffi, Federacão Brasileira de Instituicões Filantrópicas de Apoio à Saúde da Mama; **Carlos H. Barrios**, Pontificia Universidade Católica do Rio Grande do Sul School of Medicine, Porto Alegre; **Rafael Kaliks**, Hospital Israelita Albert Einstein; **Gustavo dos Santos Fernandes**, Hospital Sírio-Libanês, São Paulo; Gilberto Amorim, Centro de Oncologia D’Or, São Cristóvão; **Carlos Sampaio**, Clinica Assistência Multidisciplinar em Oncologia, Salvador; and **Gustavo dos Santos Fernandes**, Sociedade Brasileira de Oncologia, Belo Horizonte, Brazil.

## Abstract

**Purpose:**

Patients with human epidermal growth factor receptor 2 (HER2) -positive metastatic tumors treated in the public health system in Brazil do not have access to trastuzumab. This study aimed to estimate the impact of the lack of access to anti-HER2 therapies on the mortality of these patients.

**Methods:**

On the basis of published data, the number of patients with HER2-positive advanced breast cancer in 2016 who should receive anti-HER2 targeted therapy was estimated. Three different treatment groups were considered for this hypothetical cohort: chemotherapy alone, chemotherapy plus trastuzumab, and chemotherapy plus trastuzumab and pertuzumab. The number of patients alive after 2 years of follow-up was estimated on the basis of the efficacy results of the pivotal trials considering these interventions.

**Results:**

It was calculated that 2,008 women will be diagnosed with advanced HER2-positive breast cancer in Brazil in 2016. It was estimated that only 808 women would be alive in 2018 if they receive only chemotherapy (which is the treatment offered by the public health system). On the other hand, the bar rises to 1,408 women alive in 2018 if they receive chemotherapy plus trastuzumab and 1,576 women alive in 2018 if they receive the gold standard of chemotherapy plus trastuzumab and pertuzumab.

**Conclusion:**

Trastuzumab is included in the WHO’s list of essential medications, but the Brazilian public health system does not yet provide this treatment to its population with advanced disease. The introduction of trastuzumab and pertuzumab would have a positive effect, preventing premature deaths in women with metastatic HER2-positive breast cancer in Brazil.

## INTRODUCTION

Breast cancer is the most common cancer in women worldwide, and 70% of breast cancer deaths occur in women from low- and middle-income countries.^1^ Brazil has a population with diverse ethnic, cultural, and socioeconomic backgrounds, and provision of health care throughout the country is a major challenge. Cancer is the second leading cause of death in the country after cardiovascular disease.^2^ The Brazilian National Cancer Institute (INCA) estimates there will be 57,960 new cases of breast cancer in 2016,^3^ and approximately 15,000 Brazilian women die as a result of breast cancer each year.^4^ Access to health care delivery is associated with cancer outcomes.^5^

Roughly 15% to 20% of human breast cancers are classified as human epidermal growth factor receptor 2 (HER2) positive, a subgroup of tumors with a more aggressive clinical course and worse prognosis. The discovery of HER2 led to the development and approval of the first HER2-targeted therapy, trastuzumab.^6^ It is now clear that the advent and routine use of targeted anti-HER2 therapies have dramatically improved disease control and survival in patients with HER2-positive breast cancer. The list of essential medicines of the WHO includes trastuzumab both for early-stage as well as for metastatic HER2-positive breast cancer.^7^ The world cancer nongovernmental community, represented at World Health Assembly by the Union for International Cancer Control board members, is playing a key role in decreasing inequity in health care services. For several years, national advocacy patient coalitions, through collaborative initiatives, have been trying to influence public policies for the access to innovative therapies for patients with advanced breast cancer, with no success.^8^

Within Brazil’s public health system, which covers approximately three-fourths of the country’s population, access to HER2-targeted therapy is restricted. The current internationally accepted standard of care for first-line therapy in metastatic HER2-positive breast cancer is trastuzumab plus pertuzumab plus chemotherapy. Up to 2 years ago, the standard had been trastuzumab plus chemotherapy for more than a decade. In Brazil, trastuzumab has been available for the adjuvant treatment of patients with early-stage disease in the public health system only since 2013, but it is yet to be offered for the treatment of women with advanced disease. Patients with private insurance (including one-fourth of the population) do have access to all approved anti-HER2 agents available, which include trastuzumab, pertuzumab, trastuzumab emtansine (T-DM1), and lapatinib. Lack of access to effective therapy is a common issue in low- and middle-income compared with high-income countries. The consequences of this discrepancy can be estimated.

This study aimed to estimate the impact of the lack of access to anti-HER2 therapies on the mortality of patients with advanced HER2-positive breast cancer treated in the public health system in Brazil.

## METHODS

The statistics published by INCA^3^ were used to define the incidence of breast cancer in Brazil. According to official information from the Instituto Brasileiro de Geografia e Estatística,^9^ 73.7% of the population is covered under the public health system, and 27.3% has some form of private insurance.

On the basis of the findings of the epidemiologic study AMAZONE,^10^ we assumed that approximately 20% of patients have HER2-positive disease and that 94% present with stages I to III, whereas 6% are diagnosed with stage IV disease. Assuming a constant incidence within the last 5 years, we calculated the number of women diagnosed with metastatic breast cancer in 2016 as the sum of the de novo diagnoses in 2016 plus the estimated recurrences in patients diagnosed in the last 5 years. Recurrence rates for patients diagnosed with stages I to III HER2-positive breast cancers treated with chemotherapy plus trastuzumab were calculated based on published data from the HERA trial.^11,12^ Although in the public health system, trastuzumab has only been used for early-stage disease since 2013, we assumed a conservative recurrence rate as if all patients had received trastuzumab-based (neo) adjuvant therapy.

We considered three potential treatment groups for this analysis. Patients could be treated with chemotherapy alone, chemotherapy plus trastuzumab, or chemotherapy plus trastuzumab and pertuzumab.

Survival data for the chemotherapy alone arm were based on the results of the randomized clinical trial published by Slamon et al^6^ in 2001. After the publication of this trial, the use of chemotherapy alone was no longer included as part of any randomized clinical trial in this setting because it was considered unethical in view of the superiority of the trastuzumab-containing arm. Survival data for the other two groups, chemotherapy plus trastuzumab versus chemotherapy plus trastuzumab and pertuzumab, were based on the results of the CLEOPATRA trial.^13^

## RESULTS

### Number of Diagnoses of HER2-Positive Metastatic Breast Cancer in Brazil in 2016

INCA estimates there will be 57,960 new cases of breast cancer in 2016, of which 73.7% (42,717) will be covered by the public health system. According to the AMAZONE trial, roughly 20% of these cases are HER2-positive (8,543), of which 94% (8,030) present with stages I to III disease and 6% (513) with stage IV.

We estimated that 2,008 women would be diagnosed with HER2-positive metastatic breast cancer in 2016. This number was obtained by adding the patients presenting with stage IV disease in 2016 (513 women) to those developing a recurrence of the disease after receiving (neo) adjuvant treatment (1,495 women) in the previous 5 years. The number of recurrences within the last 5 years was estimated on the basis of the results of the HERA trial.^11,12^ These data are summarized in Table 1.

**Table 1:**
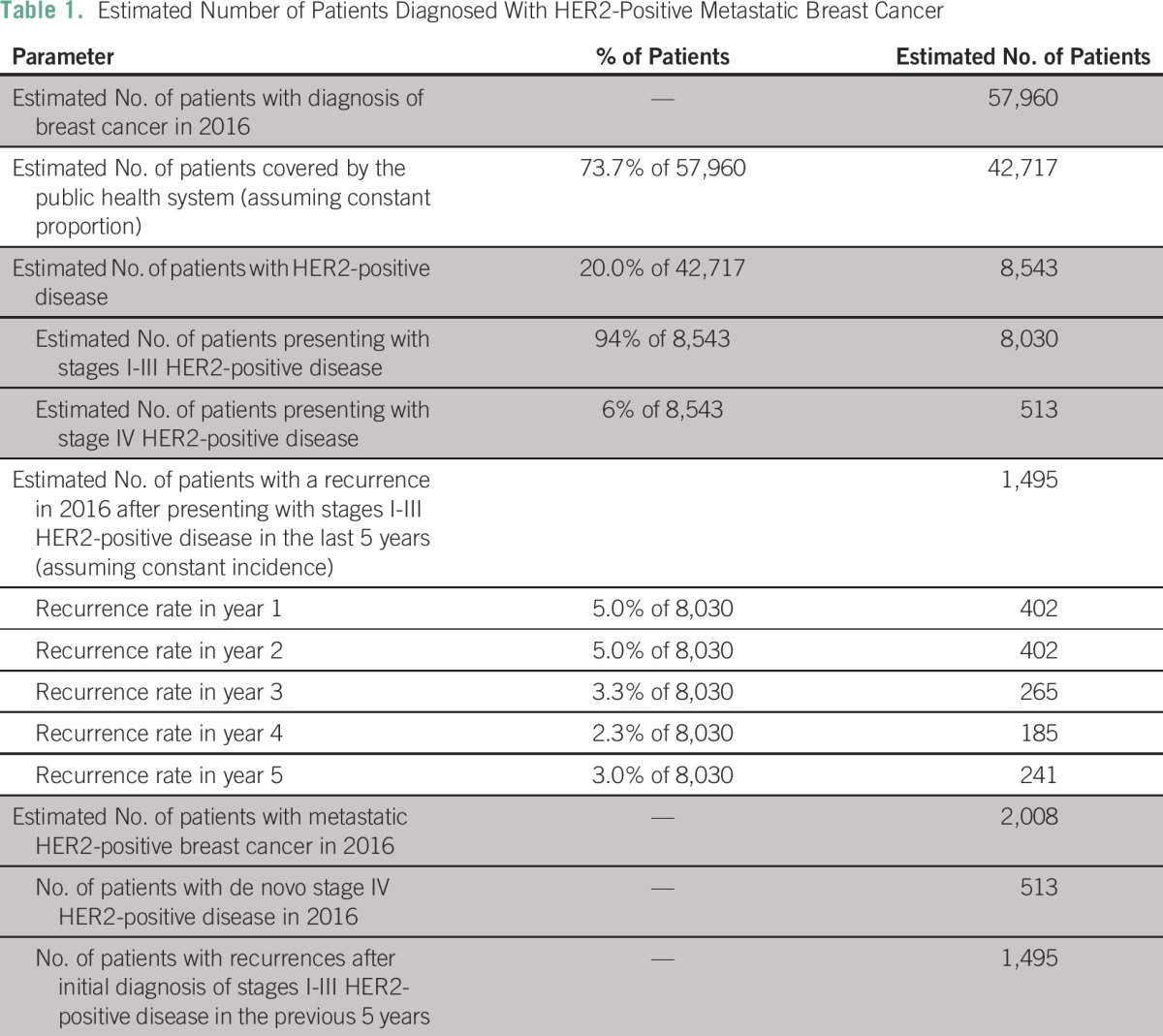
Estimated Number of Patients Diagnosed With HER2-Positive Metastatic Breast Cancer

### Estimating Survival for the Treatment Groups

On the basis of the results of the CLEOPATRA trial^13^ and the trial published by Slamon et al^6^ in 2001, median overall survival for the treatment groups was estimated as follows: 20.3 months for chemotherapy alone; 40.8 months for chemotherapy plus trastuzumab; and 56.5 months for chemotherapy plus trastuzumab and pertuzumab.

Considering 2,008 women with metastatic HER2-positive breast cancer in 2016 and on the basis of the median overall survival results, mortality rates of 50, 25, and 18 deaths per month were estimated for the treatment arms chemotherapy alone, chemotherapy plus trastuzumab, and chemotherapy plus trastuzumab and pertuzumab, respectively.

Applying these monthly mortality rates to the next 24 months, we estimated that of the 2,008 women with metastatic disease in 2016, 808 women would be alive after 2 years of follow-up if treated with chemotherapy alone. If these same women were treated with chemotherapy plus trastuzumab (as in the control arm of the CLEOPATRA trial), the number of patients alive after 2 years would increase to 1,408. This represents an absolute gain of 600 more women alive after 2 years as the result of treatment with trastuzumab. Furthermore, treatment with chemotherapy plus trastuzumab and pertuzumab would result in a total of 1,576 women alive after 2 years, an absolute gain of 768 patients. Table 2 summarizes these data.

**Table 2:**
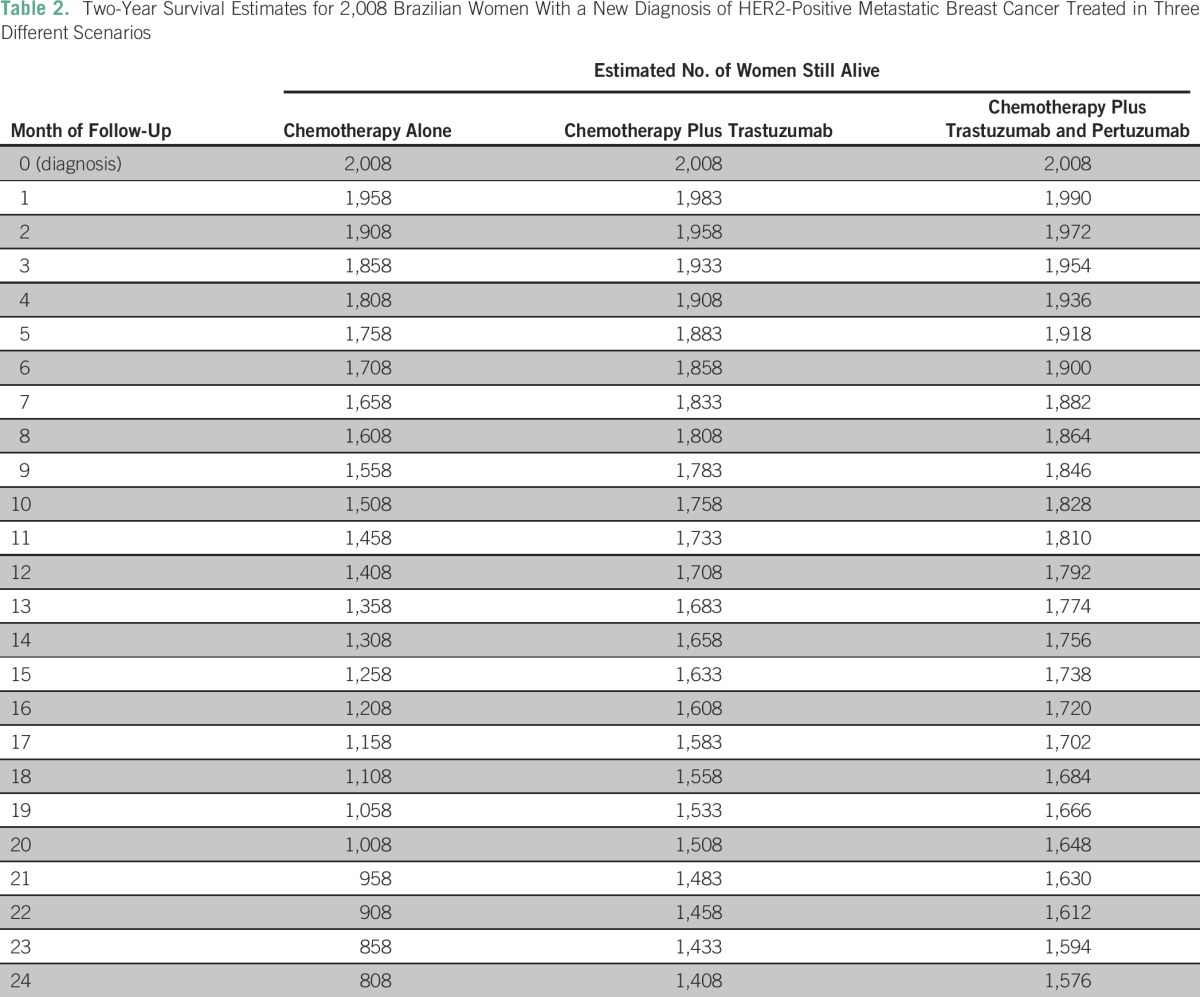
Two-Year Survival Estimates for 2,008 Brazilian Women With a New Diagnosis of HER2-Positive Metastatic Breast Cancer Treated in Three Different Scenarios

## DISCUSSION

Major efforts and achievements in medical research in the last decades have led to a better understanding of the complex molecular heterogeneity of cancer in general and breast cancer in particular. Arguably, the concept of heterogeneity is one of the important contributions to our approach to the disease. The discovery of the HER2 alteration^14^ as a driver of disease biology in up to one-quarter of breast cancers represents a paradigm shift in the way this malignancy is understood. It is now widely recognized that breast cancer comprises heterogeneous subtypes that require different treatment approaches.^15^ The successful development of trastuzumab, the first drug to target HER2-positive cancer, validates the concept that we can improve outcomes by treating an underlying molecular driver. In the past decade, three other HER2-directed therapies—lapatinib, pertuzumab, and T-DM1—have earned regulatory approval based on consistent and reproducible data indicating benefits both in the metastatic and early-disease settings.^14,16^

Several studies have identified HER2-positive breast cancers as having a more aggressive biology and being associated with inferior outcomes, including shorter time to disease progression and shorter overall survival.^17,18^ The advent of anti-HER therapies has significantly overridden this poor prognosis, so that patients with HER2-positive metastatic breast cancer treated with modern HER2-targeted therapies now experience outcomes comparable, if not superior, to their counterparts with HER2-negative disease.^19^

Pivotal trials illustrate the evolution of the treatment of advanced HER2-positive breast cancer in terms of overall survival benefits. In the late 1990s, patients who were randomly assigned to chemotherapy had a median overall survival of 20.3 months.^6^ More recently, patients treated with chemotherapy and trastuzumab in the CLEOPATRA trial had a median overall survival of > 40 months. In the same trial, patients who received double anti-HER blockade with pertuzumab and trastuzumab in combination with taxane chemotherapy demonstrated an overall survival of > 56 months.^13^ These clinically significant improvements in outcomes have been associated with acceptable toxicity profiles, as demonstrated by patient-reported outcomes from randomized clinical trial data. In particular, long-term administration of anti-HER2 monoclonal antibodies has been found to be much more tolerable than protracted chemotherapy. Routine follow-up of cardiac function, albeit the most serious potential adverse event, leads to early detection of cardiac dysfunction, allowing for appropriate and safe management of most affected patients. Recognizing these significant benefits, the WHO included trastuzumab in its list of essential oncology medicines.

Five-year breast cancer survival rates vary from approximately 80% in high-income countries to 60% in middle-income countries and 40% in low-income countries.^5,20^ Among other factors, financial status and access to health care delivery are associated with cancer outcomes. Striking income disparities are identified in Brazil. It is understandable that wealth distribution in the country affects the distribution of disease, access to health care, and consequently the overall health and disease-specific survival rates of the population. Several factors that explain some of these disparities in outcome have been previously analyzed. These include delays in diagnosis as a result of low awareness of cancer and implementation of mammography screening, and treatment-related issues such as quality of surgery as well as limited access to radiotherapy and modern systemic therapies.^1^

The Brazilian health system has two major components: the public system, Sistema Único de Saúde (SUS), with funding provided by the Ministry of Health, and the supplemental or private system, comprising voluntary and noncompulsory health plans and insurances. Approximately 73% of the population has access to health care only through SUS.^1,21^

For a new drug to be approved in Brazil, it must follow a strict regulatory process coordinated by the Agencia Nacional de Vigilância Sanitária (ANVISA).^22^ Once registration is granted through this process, most drugs (with few exceptions) become available to patients in the private health system. For patients in the public system, however, an additional approval step where cost-effectiveness analysis is performed by another commission, Comissão Nacional de Incorporação de Tecnologias (CONITEC), is necessary. Most modern high-cost medications evaluated by CONITEC have not been approved for use by patients in the public system on account of allegedly insufficient scientific evidence of benefit, lack of cost effectiveness, or incomplete documentation on the request file.

Anti-HER2 medications are a clear example of such misguided decisions. Patients in the private system have had access to trastuzumab (since 1999), lapatinib (since 2007), pertuzumab (since 2013), and T-DM1 (since 2014). In the public system, patients with early-stage HER2-positive breast cancer have had access to trastuzumab for only the last 3 years; those with metastatic disease still do not receive any form of targeted anti-HER2 therapy, even though reproducible and consistent benefits have been clearly demonstrated for almost two decades. Estimation of the number of deaths as a consequence of the late introduction to adjuvant trastuzumab has previously been presented.^23^

This enormous gap in the quality of breast cancer treatment between public and private settings is yet another cause of the significant disparities on top of late diagnosis,^22^ delays in treatment, and insufficient radiotherapy. In 2006, women with HER2-positive breast cancer were 10 times more likely to receive trastuzumab if they were privately insured. At the time, only 6% of patients with HER2-positive breast cancer received trastuzumab in the public system versus 56% in the private sector.^1,24^ The more advanced stage at diagnosis, coupled with insufficient access to modern systemic therapies, dooms patients to a worse survival outcome when compared with those with private health coverage.

One alternative patients have sought while facing these restrictions to access is to file specific lawsuits against the government to receive trastuzumab, a common situation in almost all Latin American countries.^25^ The legal basis for these litigations is the Brazilian constitution, which guarantees universal medical care to all Brazilian citizens.^26^ A second partial solution has been found by some state governments, who supplement the list of cancer therapies with a few high-cost modern therapies (including trastuzumab). These local solutions end up being only temporary and partial.

Although trastuzumab received ANVISA’s approval for treatment of metastatic HER2-positive disease in 1999, it still has not become available for patients in the public system. Its approval for the treatment of early disease was granted in 2007. Patients with private insurance have had access to treatment since that date, but those with public coverage have only had access to trastuzumab in the adjuvant setting since the end of 2012.^27^ The lack of access to potentially beneficial treatment has consequences that can be estimated.

Our results show that approximately 2,008 women will be diagnosed with metastatic HER2-positive breast cancer in 2016. On the basis of our estimates, if treated with chemotherapy without any HER2-targeted agents, only 808 of these women will be alive in 2018. On the other hand, if trastuzumab is incorporated, 1,408 of them are expected to be alive. The availability of trastuzumab in the public health system for patients with metastatic disease would result in an additional 600 women alive at the end of 2 years. Making this situation even more dramatic, the publication of the CLEOPATRA trial^13^ led to a change in the gold standard treatment of HER2-positive advanced breast cancer: dual blockade with trastuzumab and pertuzumab. According to our analysis, if dual HER2 blockade plus chemotherapy were used, the difference in the number of women alive after 2 years would be even higher: 768 patients.

We acknowledge that delivering on the promise of precision medicine to all patients will require a massive effort, not only from doctors and researchers, but also from pharmaceutical companies and governmental agencies, among others. At the same time, it is imperative to recognize that the consequences of depriving a significant proportion of patients from low- and middle-income countries of effective therapy can be estimated. This exercise should be an important component to be taken into account when discussing what will or what will not be offered as treatment.

This analysis has significant limitations. Specific data from low- and middle-income countries are sparse and under-reported, meaning we had to rely mostly on estimates from INCA. Additionally, it is important to state that we are extrapolating survival differences directly from randomized clinical trials, and it is possible that these differences are less pronounced in general clinical practice. This assumption might introduce a bias favoring the difference between treatments in real life. On the other hand, because trastuzumab was introduced in the adjuvant treatment only in 2013, our estimates are conservative by assuming a lower relapse rate than that of patients who do not receive trastuzumab in this setting. In conclusion, 15 years after the worldwide approval of trastuzumab for metastatic breast cancer and 1 year after its inclusion in the WHO essential medicines list, the Brazilian public health system does not yet provide this treatment to its population. The introduction of trastuzumab and pertuzumab would significantly increase the life span of women with metastatic HER2-positive breast cancer in Brazil and prevent 768 premature deaths in the next 2 years.
